# Gender differences in influences of temperament on olfactory reactivity and awareness

**DOI:** 10.1038/s41598-017-09231-z

**Published:** 2017-08-21

**Authors:** Lenka Martinec Nováková, Radka Vojtušová Mrzílková, Anna Kernerová

**Affiliations:** 1 Department of Anthropology, Faculty of Humanities, Charles University, U Kříže 8, Prague 5 - Jinonice, 15800 Czech Republic; 2 National Institute of Mental Health, Topolová 748, 25067 Klecany, Czech Republic

## Abstract

Children’s olfactory performance is associated with temperament but whether there is a link with olfactory reactivity and awareness is not known. In adults negative affectivity is linked to reactivity to environmental odours but it is not clear whether these associations extend to children. We aimed to investigate the effect of temperamental factors on olfactory reactivity and awareness. In so doing, we controlled for the effect of parenting styles on temperamental assessment and of verbal fluency on children’s olfactory reactivity and awareness. We hypothesised that children with a high degree of negative affectivity would show greater olfactory reactivity and awareness. 129 children (62 boys, mean age 6.83 ± 0.40 years) were interviewed about their olfactory reactivity and awareness in everyday life using the established Children’s Olfactory Behavior in Everyday Life questionnaire (COBEL). Parents assessed their child’s temperament using the 94-item short form of the Children’s Behavior Questionnaire. We found that the relationship between negative affectivity and total COBEL scores varied between the genders: there was a positive, medium to large effect in boys and a negative, small one in girls. Future studies could include behavioural observations of temperament and olfactory reactivity and awareness.

## Introduction

A high degree of interindividual variability in olfactory perception has been reported, particularly in olfactory abilities^1^ and various olfaction-related metacognitive measures^2^. The latter lend insight into people’s interactions with their everyday olfactory environments, which are not directly observable or reproducible in the laboratory. Termed “odour awareness”^2, 3^ or “olfactory reactivity and awareness”^4, 5^, this metacognitive aspect of olfactory perception encompasses the degree to which individuals pick up olfactory stimuli in their vicinity, let them affect their attitudes and behaviours, seek pleasant olfactory stimulation and avoid unpleasant, potentially disturbing or offensive odours. Differences in olfactory reactivity and awareness are apparent from a young age^4, 6, 7^.

Variability in the ways in which olfactory environment is experienced has been related to various between- and within-gender differences^3, 8–10^. One of the probably relatively less influential factors in general, which nevertheless deserves attention, is personality or temperament. Links have been sought particularly with the personality trait of neuroticism or its subscale, anxiety, for an overview see Martinec Nováková and Vojtušová Mrzílková^11^. The rationale behind such studies is that amygdala and hippocampus, which are involved in both olfactory^12^ and emotional processing^13^, exhibit structural and functional changes in anxious persons^14^, with possible implications for the olfactory domain. The evidence available indeed seems to suggest differences in  olfactory performance in people relatively higher on neuroticism and anxiety, see^11^ for an overview of adult literature. However, little is known about whether such effects also pertain to the metacognitive aspects of olfaction, namely olfactory reactivity and awareness. Smeets, *et al*.^2^ reported that awareness of odours to be avoided was linked to experiencing health effects attributed to environmental odours, which, in turn, was associated with negative affectivity. Negative affectivity appears to influence reactivity to environmental odours and reports of subjectively assessed exposure-related olfactory and respiratory symptoms^15, 16^. In line with this is the finding that highly neurotic individuals respond more negatively to environmental odours^17^. Buron *et al*.^18^ also reported a link between the Relational Scale of Olfaction (EROL) and anxiety. On the other hand, Seo, *et al*.^19^ did not show any associations between neuroticism and attitudes towards olfaction.

Another research gap is the lack of studies in individuals of a wider age range. As can be seen from the overview of literature on links between personality and olfaction^11^, participants in most of the studies were young adults, predominantly university students, which raises the question as to whether the links between personality and olfaction exist outside this particular age range and social background. Due to the cumulative, potentially much stronger effects of physiological aging, lifestyle, medical history, and environmental exposure on chemosensory perception, which are likely to obscure the effect of personality and temperament in adults, a somewhat more attractive idea is to turn to children. As in adults, in children such a research topic is justified by the fact that associations between alterations of the limbic system and trait anxiety and fearfulness have been observed in young healthy children^20^. One would expect that if these changes have implications for emotional as well as olfactory processing, differences in olfaction among children with varying levels of anxiety should be seen. They should stem from general, constitutionally based tendencies to react in a certain way, which (unlike personality) can be assessed at a very young age. Such tendencies may be subsumed under the term temperament.

Indeed, in adults, individual differences have generally been conceptualized in terms of personality theory and traits, whilst in young children, temperamental measures have been employed. Since personality is formed gradually through experience as the child gets older, there is little consensus as to whether it is meaningful to measure personality in young children. Related to this, no single model of personality has been established as the most useful one in young children thus far, even though some researchers report that there are correlations between temperament and personality in children^21^. Their findings are, in general, in line with mappings between temperament and personality in adults. In short, the set of temperamental traits underlying neuroticism, referred to as negative affectivity, reflects, in general, threat sensitivity with accompanying negative affect. Behaviourally it is manifested in anxious and fearful reactions, inhibition of ongoing behaviour, increased readiness for action, and heightened attention to environmental stimuli^22^. Although the factor of negative affectivity seems to map fairly well onto the Big Five^23^ dimension of neuroticism^24^, its expression is substantially modulated by self-regulatory processes subsumed under effortful control. This refers to a self-regulatory system superordinate to it that manifests itself in the ability to voluntarily sustain focus on a task, shift attention from one task to another, initiate action, and inhibit it^25^. The set of temperamental traits underlying extraversion, termed surgency, encompasses sensitivity to signals of reward and active engagement with the surrounding world. It is manifested behaviourally through sociability, impulsivity, sensation seeking, and activity level, and partly also via intensity of affective response, in particular positive affect^22^. Surgency maps quite well onto extraversion, with its expression also being modulated by effortful control^24^.

Research in adults has suggested a link between olfactory perception and personality. However, it is not clear whether these relationships extend beyond this particular age group and whether they involve olfactory reactivity and awareness. The aim of the study was to investigate the effect of temperamental factors on olfactory reactivity and awareness in children. In so doing, we controlled for the effect of parenting styles on temperamental assessment by parents and of verbal fluency on children’s olfactory reactivity and awareness. To be specific, since neuroticism and negative affectivity (which maps broadly onto neuroticism) appear to heighten olfactory reactivity and awareness in adults, we hypothesized that children deemed to exhibit a high degree of negative affectivity would show greater olfactory reactivity and awareness. Furthermore, since children’s olfactory reactivity and awareness have been shown to differ in boys and girls^4^, as a subsidiary aim, we tested for gender differences.

## Results

### Exploratory Correlations

Descriptive statistics are shown in Table 1 and exploratory Spearman’s ρ rank correlations are given in Supplementary Table S1. Children’s total COBEL scores were not significantly linked to any of the temperamental factors, but the social component of COBEL correlated with effortful control, ρ = 0.198, p = 0.02, Cohen’s d = 0.46, and the environmental component was associated with negative affectivity, ρ = 0.176, p = 0.047, Cohen’s d = 0.40. The latter was also found in boys, ρ = 0.264, p = 0.038, Cohen’s d = 0.63, but not in girls. Children’s olfactory reactivity and awareness did not vary with respect to their age or verbal fluency, ρ = 0.08, p = 0.35, Cohen’s d = 0.12, and ρ = 0.15, p = 0.10, Cohen’s d = 0.12, respectively. Parenting styles were not significantly associated with any of the three broad temperamental factors (ρ*s* ranging between −0.140 and 0.133, p*s* > 0.1). Therefore, only the effects of temperament, gender, and their interactions on total COBEL scores were modelled in the following analyses.

**Table 1:** Descriptive statistics for boys, girls, and the total sample, Mann-Whitney U for differences between boys and girls, p value, and effect size *r*, which was converted to Cohen’s *d*.

	Mean ± SD			Mann-Whitney U	p	r (d)
	boys	girls	total			
Age	6.85 ± 0.42	6.81 ± 0.38	6.83 ± 00.40	1938.50	0.514	−0.06 (−0.12)
Verbal fluency	12.76 ± 4.37	13.90 ± 4.02	13.35 ± 4.21	2427.50	0.097	−0.14 (−0.28)
COBEL total	5.60 ± 2.45	6.75 ± 2.20	6.20 ± 2.39	1485.50	**0.005**	−0.25 (−0.52)
COBEL food component	1.27 ± 0.79	1.47 ± 0.68	1.38 ± 0.74	1798.50	0.181	−0.12 (−0.24)
COBEL social component	1.25 ± 0.97	1.70 ± 0.97	1.48 ± 0.99	1551.00	**0.012**	−0.22 (−0.45)
COBEL environmental component	3.07 ± 1.32	3.58 ± 1.27	3.34 ± 1.32	1624.00	**0.032**	−0.19 (−0.39)
Surgency	4.57 ± 0.93	4.37 ± 0.91	4.46 ± 0.92	1822.50	0.230	−0.11 (−0.22)
Negative affectivity	3.97 ± 0.73	3.88 ± 0.70	3.92 ± 0.72	1850.50	0.286	−0.09 (−0.18)
Effortful control	4.94 ± 0.66	5.42 ± 0.56	5.19 ± 0.65	1188.50	**<0.001**	−0.37 (−0.80)
Parenting style – Authoritarian	54.00 ± 32.11	55.51 ± 27.49	54.78 ± 29.69	2056.00	0.921	−0.01 (−0.02)
Parenting style – Permissive	11.90 ± 14.12	16.78 ± 23.85	14.43 ± 19.86	2044.50	0.877	−0.01 (−0.02)
Parenting style – Authoritative	76.78 ± 29.67	83.37 ± 25.20	80.20 ± 27.53	1815.50	0.196	−0.11 (−0.22)

### Influence of Temperament on Children’s Olfactory Reactivity and Awareness

As can be seen from Table 2, showing categorical regressions on the total sample, Model 1 containing only the three temperamental factors was not statistically significant. Inclusion of gender and interactions thereof with each of the temperamental factors yielded significant models (Model 2 and 3), with significant R^2^ change (ΔR^2^) indicating the inclusion significantly improved the prediction. However, this was not the case with Model 4. Therefore, Model 3 was chosen as the most suitable one. It explained 14.5% of variation in olfactory reactivity and awareness in the present sample, with an estimated 9.6% in the population, which is slightly above the recommended minimum effect size for representing a “practically” significant effect for social science data^26^. Most importantly, it showed that although there was no main effect of any of the three temperamental factors, the interaction variable of gender*negative affectivity was a significant predictor of total COBEL scores. That is, the effect of negative affectivity on total COBEL scores varied between the genders, β = 0.669, p = 0.034.

**Table 2:** Categorical regression (CATREG) models in the total sample regressing the total COBEL scores on the three temperamental factors (Model 1), gender (Model 2), interaction of temperament and gender (Model 3), and interactions among the temperamental factors (Model 4).

	Model 1	Model 2	Model 3	Model 4
R^2^	0.043	0.084	0.145	0.156
R^2^ (adj.)	0.020	0.055	0.096	0.085
*f* ^2^	0.04	0.09	0.17	0.18
F	1.875	2.859	2.933	2.185
p	0.137	**0.026**	**0.007**	**0.023**
ΔR^2^	0.043	0.041	0.061	0.011
p		**0.020**	**0.039**	0.674
Surgency	0.110	0.104	0.424	0.525
p	0.215	0.257	0.114	0.114
Negative affectivity	0.144	0.141	−0.491	−0.492
p	0.174	0.188	0.100	0.163
Effortful control	0.201	0.118	0.204	0.211
p	0.022	0.233	0.476	0.512
Gender		0.219	0.213	0.217
p		**0.021**	0.025	**0.028**
Gender*Surgency			−0.314	−0.436
p			0.270	0.198
Gender*Negative Affectivity			0.669	0.650
p			**0.034**	0.076
Gender*Effortful Control			−0.059	−0.082
p			0.849	0.798
Surgency*Negative Affectivity				0.093
p				0.358
Surgency*Effortful Control				−0.037
p				0.717
Negative Affectivity*Effortful Control				−0.024
p				0.834

Gender is coded as 0 = boy and 1 = girl. The predictor-related values represent β*s*. Significant effects to be interpreted are highlighted in bold. The model that seems to provide the best explanation is Model 3.

As can be seen from Supplementary Table 1, in boys the link between negative affectivity and olfactory reactivity and awareness was positive, although marginally significant, Spearman’s ρ = 0.247, p = 0.053, Cohen’s d = 0.62. This represents a medium to large effect and can be interpreted in two ways. According to Cohen^27^, an effect size of 0.6 indicates that the mean olfactory reactivity and awareness of boys high on negative affectivity was at about the 73^rd^ percentile of those low on the trait. Alternatively, it suggests that there was about a 38% non-overlap in the distribution of COBEL scores of boys high and low on negative affectivity, respectively. On the other hand, in girls the association was negative and non-significant, ρ = −0.018, p = 0.887, Cohen’s d = −0.18, indicating that the mean olfactory reactivity and awareness of girls low on negative affectivity was at about the 58^th^ percentile of those high on the trait. Alternatively, the non-overlap between the distributions of COBEL scores was only about 15% in girls. When the total COBEL scores were then regressed on temperamental measures in boys and girls, respectively (see Table 3), it transpired that in boys, the model explained 10.8% of variation in olfactory reactivity and awareness in boys, with an estimated 6.2% in the population, although it missed significance at p = 0.083. Again, this effect size represents a “practically” significant effect for social science data^26^. However, this was not the case in girls, as there were only 5.5% of variation in olfactory reactivity and awareness explained by the model, with an estimated 1% in the population (p = 0.308). In sum, the effect size of the relationship between negative affectivity and olfactory awareness and reactivity seemed relatively larger in boys than in girls and of “practical” relevance in the former but not in the latter, although, with the present sample size, p-values well below the conventional 0.05 level were not seen.

**Table 3:** CATREG models in boys (Model 5) and girls (Model 6) regressing the total COBEL scores on the three temperamental factors. Df_1_ and df_2_ denote regression and residual degrees of freedom, respectively. The predictor-related values represent β*s*.

	Boys (Model 5)	Girls (Model 6)
R^2^	0.108	0.055
R^2^ (adj.)	0.062	0.01
*f* ^2^	0.12	0.06
F(df_1_,df_2_)	2.339 (3,58)	1.225 (3,63)
p	0.083	0.308
Surgency	0.034	0.225
p	0.821	0.078
Negative Affectivity	0.353	−0.055
p	0.027	0.690
Effortful Control	0.152	0.125
p	0.294	0.298

### Between-Gender Variation in Temperament, Verbal Fluency, and Parenting Styles

As can be seen from Table 1, girls and boys did not produce significantly different words counts per minute. Girls were thought to exhibit greater effortful control, but not negative affectivity or surgency. As reported previously^11^, they were rated higher on all of the constituent subscales of effortful control. Furthermore, girls were more likely than boys to exhibit sadness, whilst boys showed a greater degree of anger and frustration^11^. Ratings of agreement with the hypothetical responses portrayed by the vignettes representing parenting styles did not differ depending on whether the child was a boy or a girl.

## Discussion

The aim of the present study was to investigate the influence of parental assessments of children’s temperament on their self-reported olfactory reactivity and awareness, operationalized in terms of COBEL scores. We hypothesised that children deemed by their parents to show a greater degree of negative affectivity would show greater olfactory reactivity and awareness. We found that the effect of negative affectivity on total COBEL scores varied between the genders. Overall, it transpired that the effect size of the relationship between negative affectivity and olfactory awareness and reactivity was relatively larger in boys than in girls and of “practical” relevance in the former but not in the latter, even though, with the present sample size, statistical significance in the conventional terms of p < 0.05 was not obtained. As statistical significance largely depends on sample size, e.g. Cumming^28^, here we focus on interpretation of the effect sizes found. First of all, the findings in boys are partly in line with the findings reported by Smeets, *et al*.^2^, who found a link in adults between awareness of odours (specifically those to be avoided) and experiencing health effects attributed to environmental odours, which was linked to negative affectivity. Dalton also^29^ describes the results of several studies^30–32^ investigating the effects of negative affectivity on perception of and reaction to ambient exposures to acetone and isopropyl alcohol (IPA; rubbing alcohol). For instance, when exposed to acetone at concentrations well within exposure limits, individuals exhibiting greater negative affectivity reported significantly higher levels of alleged health symptoms such as sensory irritation compared to participants relatively lower on this temperamental/personality characteristic. Also, participants showing greater negative affectivity rated IPA as more unhealthy, hazardous, and annoying (but not more intense or unpleasant) than those with low negative affectivity levels. The former group reported increased health symptoms as well. Another evidence that negative affectivity influences olfactory reactivity comes from Ihrig, *et al*.^16^. The authors found that individuals with high negative affectivity levels reported significantly more alleged olfactory and respiratory symptoms upon exposure to ammonia. Finally, highly neurotic individuals respond more negatively to environmental odours^17^. Altogether these studies suggest that negative affectivity seems to play a role in the way individuals experience their olfactory environment. Our results in boys are, to some extent, in line with those previously reported in adults, including the finding that the effect sizes found in them were relatively modest but still of practical significance. It is difficult to tell what effect sizes to expect based on the previous studies in adults, but, for instance, converting correlation coefficients to R^2^ would give an effect size ranging between 0.005 and 0.14 for links between negative affectivity and irritative, respiratory, and olfactory symptoms in Ihrig^16^. If the same is done with Spearman’s ρ for the link between boys’ negative affectivity and self-reported olfactory reactivity and awareness, an R_s_^2^ of 0.06 is obtained, which places the effect found in the present study well in the middle of the range reported in adults. Even though direct comparison of R^2^ and R_s_^2^ coefficients is not possible and different designs and methods were used in the studies, clearly the amount of variance shared by olfactory reactivity and awareness on the one hand and temperament on the other only reaches about 15% at maximum.

Secondly, it is difficult to tell what the cause of different effect sizes in boys and girls, respectively, might have been. As seen in adult research not only on negative affectivity, but also anxiety and neuroticism, it is possible that the sought effect indeed is stronger in one gender than in the other, e.g.^33, 34^, although the causes for this are not known. An alternative explanation might be that in boys, reports of disturbing, unpleasant odours might have primarily contributed to higher COBEL scores, whilst girls might have reported pleasant and unpleasant odours in equal parts or focus on the former. Since individuals with high negative affectivity levels are more likely to report adverse effects of unpleasant odours on their well-being^2, 16, 30–32^, this might have led to the positive effect found in boys, but not in girls. Since use of the COBEL questionnaire does not allow further quantitative analysis of reports of pleasant and unpleasant odours, we have no evidence to support this idea and we present it as a suggestion for further research. On the other hand, ruled out as a potential cause can be characteristics of the present sample, such as gender differences in variation in negative affectivity or in olfactory reactivity and awareness.

Children’s olfactory reactivity and awareness showed a gender difference in favour of girls, which is in line with the study by Saxton, *et al*.^4^. However, one of the key issues when interviewing children is that they can interpret the researcher’s questions as a search for the “right answer” and thus may say what they believe he or she wants to hear. This is the case particularly in contexts in which the researcher is perceived as a person in authority and the interview is conducted at school^35^. With girls being in general more sensitive to the presence of a researcher^36^, their superior performance on the interview might have been a by-product of their greater tendency to “please” the researcher. To counteract such effects, researchers may want to exclude the children’s first answers as they may represent a response that the children believe is expected, desirable, or appropriate^37^. However, the research protocol in the present study did not allow for such an approach. Importantly, contrary to what had been proposed in the previous studies^5, 7^, gender differences in olfactory reactivity and awareness did not seem an expression of variability in verbal fluency in the present study. Girls, who have been reported to outperform boys on measures of verbal fluency^38, 39^, did not produce significantly more words per minute and, moreover, verbal fluency was not linked to olfactory reactivity and awareness.

Girls and boys were also found to differ on several temperamental traits. As discussed at length earlier^11^, such differences may possibly be a result of parents’ different approach to assessment of boys’ and girls’ temperament^40^. Parental assessments of children’s temperament may also vary depending on whether the responses are provided by the child’s mother or father^40^. This, however, did not play a major role in the present study, with 88% of temperamental assessments having been provided by mothers.

Children’s olfactory reactivity and awareness did not vary with respect to their age or verbal fluency, which were correlated, as could be expected^41^. In previous studies, children’s olfactory reactivity and awareness were found to increase with age^4, 5, 7^, which was ascribable to the children’s progressively better memory and verbal fluency. The absence of such links in the present study could be attributed to the low variability in age. Parenting styles were not significantly associated with any of the three broad temperamental factors in the total sample. As complex interactions tend to be found between parental perceptions of their children’s temperament and the parenting style employed^24, 42^, the possible explanation is that the brief assessment using hypothetical vignettes was not sensitive enough. To the best of our knowledge, at present there are only two previous studies which used this approach to assess parenting styles: the original paper by Barnhart *et al*.^43^ and the study by Martinec Nováková and Vojtušová Mrzílková^11^, in which no effect on the temperamental measures was found. Thus, there are no data available on the reliability and validity of the tool.

Finally, there are several challenges when investigating links between olfactory metacognition and temperament in children. First and foremost, both undergo developmental changes so that behaviours indicative of a certain temperamental trait or a level of olfactory awareness in younger children may not be equally informative, reliable, or valid in the older ones. In the present study, this has been overcome by employing age-specific instruments to assess temperament and olfactory metacognition^5, 44^. In spite of developmental changes, temperament is relatively stable and heterotypically continuous in that there are relations between different sets of behaviour across different ages^45, 46^ and it predicts personality in young adulthood^47^. Also, assessment of child temperament is largely dependent on other-reports, especially parental or those by teachers, which is the least time-consuming method. However, there is reasonable consistency between assessments based on home and laboratory observations on the one hand and parental reports on the other one^48, 49^. There are also significant correlations for most of the scales of self- and parental reports where both of these are available^50^. Therefore, use of other-report measures, such as parental assessment, of child temperament appears an acceptable trade-off between the opportunity to address this research question in the youngest cohorts and precision of measurement. On the other hand, to the best of our knowledge, there are no published instruments for parents or teachers with which to assess children’s olfaction-related behaviours. While correlating children’s self-reports^5^ with parental or teacher’s assessments on the one hand reduces the possibility of obtaining significant results merely due to the fact that the questionnaires have been completed by a single person, on the other their responses may be subject to bias mentioned above.

The present study addressed the research question of the effect of temperament on children’s olfactory reactivity and awareness. We hypothesised that children deemed by their parents to show a greater degree of negative affectivity would exhibit greater olfactory reactivity and awareness. We found that the effect of negative affectivity on total COBEL scores varied between the genders. The effect size of the relationship between negative affectivity and olfactory awareness and reactivity was relatively larger in boys than in girls and of “practical” relevance in the former but not in the latter. As the effect of negative affectivity on how olfactory environment is experienced seems to be mediated by the perceived characteristics of odours, future studies should investigate whether this temperamental trait affects specifically reactivity to and awareness of potentially disturbing or harmful odours. Even though parental (and teacher) assessments are largely consistent with behavioural observations of temperament, the latter could also be included in future studies. Also, given the narrow age range of children recruited in the present study, more studies with preadolescent and adolescent participants are needed to gain more insight into the nature of these relationships.

## Materials and Methods

### Participants

The participants were 129 children of Czech origin (62 boys, mean age 6.83 ± 0.40 years, range 6.11–8.25 years) and their parents (113 mothers). The children were first-graders from six public mixed-sex general education elementary schools in Prague and its suburbs. The schools were attended by children from varied social backgrounds. School principals were contacted via telephone and e-mail to inform them about the planned study. Those who had provided permission to perform the study on the school’s premises were asked to pass the information on to the teachers. They distributed leaflets to children, who, in turn, handed them over to their parents. As shown in Table 1, girls and boys did not differ in terms of age.

### Ethics Statement

All procedures followed were in accordance with the ethical standards of the responsible committee on human experimentation (institutional and national) and with the Helsinki Declaration of 1975, as revised in 2008 (5). The study was approved by the IRB of the Faculty of Science, Charles University (Approval Number 2015/11). Written informed consent was obtained from the children’s parents, and oral informed consent was provided by the children in the presence of a teacher employed by the school. The children-parents pairs each received 300 CZK (approx. 11 EUR or 12 USD) in compensation.

### Questionnaires

#### Children’s Olfactory Behaviors in Everyday Life Questionnaire (COBEL)

Olfactory reactivity and awareness was assessed by means of an interview based on the COBEL questionnaire^5^. Having been originally developed with 6- to 10-year-olds, it consists of 16 questions designed to evaluate self-reported awareness and reactivity to odours in significant everyday contexts, i.e. food, social, and environmental. Each item was coded on a 3-point scale, rating the child as poorly (0), moderately (0.5), or highly (1) olfaction-oriented in the given situation. Although it was used in a previous study with slightly older Czech children^4^, a pilot study (N = 30) described in detail elsewhere^6^ revealed that most children were unfamiliar with the rating format of Item 3 (“Senses in nature”), which involved ranking the following activities in order of preference: touching, smelling, watching, listening. Therefore, the item was excluded from the interview. Thus, the total COBEL score, computed as a sum of the 15 items, ranged from 0 to 15. In addition to the total score, component scores for food, social, and environmental odours were computed following previous usage of COBEL^4, 5, 7^, ranging from 0 to 3, 0 to 4, and 0 to 8, respectively.

#### Short Form of the Children’s Behavior Questionnaire (CBQ-SF)

Being widely used in developmental research, the Children’s Behavior Questionnaire CBQ^51^; affords a highly differentiated caregiver report assessment of temperament in three- to eight-year-olds. Domains covered by the instrument include positive and negative emotion, motivation, activity level, and attention. The short, 94-item form CBQ-SF^44^, is comprised of 15 scales of six to eight items each comprising three temperamental factors, i.e. surgency, negative affectivity, and effortful control. Parents rate their child on a seven-point scale ranging from one (extremely untrue of the child) to seven (extremely true of him or her). Temperamental factor scores were computed after Rothbart, *et al*.^52^. The questionnaire was translated to Czech by LMN and a back-translation was produced by Jitka Lindová and Jan Havlíček. The individual CBQ-SF scales and the three broader factors along with their definitions and descriptive statistics are listed in Martinec Nováková and Vojtušová Mrzílková^11^.

#### Parenting Styles

Parenting styles were briefly assessed to control for the influence of parental responsiveness and demands on temperamental attributions. Having been outlined in the seminal work of Baumrind^53^, parenting styles refer to the emotional atmosphere created by parents at home^54^. They can be classified into the following three categories: authoritative (responsiveness towards children combined with demands), authoritarian (demanding but not very responsive), and permissive (high in responsiveness and low in demands). They were briefly assessed using hypothetical vignettes after Barnhart, *et al*.^43^. These include descriptions of three hypothetical interpersonal situations between a parent and his or her two children (son and daughter) aged 7 and 11 years, respectively, in which the response of the parent to the situation represents one of the three parenting styles. For instance, in the hypothetical vignette representing the permissive parenting style, the mother believes that children should be free to do what they want, and so when the children want to go outside to play without having done their homework before, she nevertheless lets them go outside for as long as they want. The parents were asked to indicate the degree to which they associated themselves with the hypothetical parent’s response to the given situation on a 10 cm visual analogue scale. The rating for each vignette was obtained by measuring the distance in mm between zero and the marked point on the line.

### Verbal fluency

In general, interview techniques are highly dependent on the child’s verbal skills^55^ and verbal fluency has been found to be an important modulator of olfactory reactivity and awareness when using the COBEL^4, 5, 7^. This is due to the fact that some of its items are open-ended questions requiring spontaneous reports of some activity, e.g. “Imagine your parents present you with a dish you do not know: will you do something before putting it in your mouth? What do you do?”, while others rely on the child’s naming ability, e.g. “Do you remember odours you smelled yesterday? Which ones?”, which is virtually analogous to measures of verbal fluency. It was tested using a Czech version of the category verbal fluency test adapted to children^56^. First, variability of responses to the three categories (animals, fruits, and things that can be bought in a supermarket) had been explored in a pilot study described in full detail elsewhere^6^. Most variable results were obtained for animals, which were chosen as the test category. During the testing session itself, a training trial was conducted using the transport category, on which a child was asked to name as many means of transport as possible. Understanding was assured by asking the child what transport meant and by giving an example (e.g. a car). Next, the child was encouraged to name as many animals as possible in 60 seconds, while the answers were being immediately written down by the researcher.

### Procedure

The children participated in individual sessions, which were always scheduled after school dinners at a time between 12:30 p.m. and 3 p.m. (i.e. a time when most children attend after-school care centre on the school premises). Parents completed the CBQ-SF and provided ratings of the hypothetical vignettes at home via a web-based survey tool, Qualtrics (Qualtrics, Provo, UT). They were never present in the room during the session.

### Statistical Analysis

All analyses were carried out with IBM SPSS 22.0. Non-parametric analyses were preferred since some of the data (COBEL scores, parenting styles) were not normal. Gender differences were analysed using the Mann-Whitney U test and associations were tested with Spearman’s ρ rank correlations. Effect sizes for the Mann-Whitney U test were computed after Rosenthal^57^ as follows: $r=\frac{z}{\sqrt{N}}$ and then converted to Cohen’s d after Rosenthal^58^. Spearman’s ρ rank correlations were converted to Cohen’s d after Walker^59^. To test both the main effects of the temperamental variables and gender and their interactions, categorical regressions (CATREG) were run. Thanks to its optimal scaling option, this type of analysis allows exploration of the data in any way that makes sense and makes interpretation easier, regardless of their level of measurement^60^. Since the minimum scores achievable for each of the temperamental variables were greater than 0, for the purposes of meaningful interpretation of the CATREG including the intercept, the continuous variables of surgency (SY), negative affect (NA), and effortful control (EC) were centered first. This was done by subtracting the given variable’s mean from the individual values. Then, interaction variables were created by multiplying the centered temperamental variables with one another or with the dummy variable of gender. Next, a CATREG was run using the SPSS Optimal Scaling option. The assumptions of the test were met since the number of valid cases exceeded the number of predictor variables plus one. Scale variables were treated as numeric and were discretized by multiplying, nominal variables were treated as such. A random initial configuration was selected, as recommended when at least one variable is treated as nominal^61^. Perfect multicollinearity (intercorrelations >0.9) did not appear a serious problem, which was further supported by reviewing the variance inflation factors (VIF), which were nowhere near the value of 10, and the average VIF was not greater than 1, as recommended e.g. by Field^62^. The models tested were as follows: in Model 1, the total COBEL score was regressed on SY, NA, and EC; in Model 2, gender was included; in Model 3, interaction variables of SY*gender, NA*gender, and EC*gender were included, and, finally, in Model 4, interaction variables of SY*NA, SY*EC, and NA*EC were added. As for Model 4, even though both SY and NA correlated with EC (−0.5 and −0.4, respectively), as can be seen from Supplementary Table 1, multicollinearity did not seem an issue. An additional analysis to which ridge regression was applied yielded very similar results. As Model 3, which seemed to provide the best explanation of the relationship between temperament, gender, and olfactory awareness and reactivity, showed that the effect of negative affectivity on total COBEL scores varied between genders, it was followed with two other models in boys and girls, respectively. Specifically, in Models 5 (for boys) and 6 (for girls), the total COBEL score was regressed on SY, NA, and EC. The settings was the same as for the models run on the total sample, with the only exception that a numerical initial configuration was selected, as recommended when all variables are treated as numeric. Significance of R^2^ change across the models (ΔR^2^) was computed after Field^62^. Although R^2^ and adjusted R^2^ themselves represent measures of effect size, as do betas, for convenience, R^2^s were converted to Cohen’s f^2^ after Cohen^27^.

## Electronic supplementary material

Supplementary Table S1

Supplementary Dataset S2
