# Supplementary material for: Gender differences in influences of temperament on olfactory reactivity and awareness
Source: Sci Rep. 2017 Aug 21;7:8920. doi: 10.1038/s41598-017-09231-z (PMC5566423; doi:10.1038/s41598-017-09231-z)
Supplement: Supplementary file 1 — Supplementary Table S1 [file 41598_2017_9231_MOESM1_ESM.pdf]

Gender differences in influences of temperament on olfactory reactivity and awareness

Lenka Martinec Nováková[a, b*], Radka Vojtušová Mrzílková[a] and Anna Kernerová[a, b]

[a]*Department of Anthropology, Faculty of Humanities, Charles University, U Kříže 8, 158 00 Prague 5 – Jinonice, Czech Republic*

[b]*National Institute of Mental Health, Topolová 748, 250 67 Klecany, Czech Republic*

* Correspondence to be sent to: Lenka Martinec Nováková, Department of Anthropology, Faculty of Humanities, Charles University, U Kříže 8, 158 00 Prague 5 – Jinonice, Czech Republic. Email: lenka.novakova@fhs.cuni.cz

Supplementary Table S1. Exploratory Spearman's rho rank correlations between COBEL scores (total and components), temperamental factors, age, verbal fluency, and parenting styles in the total sample (N = 129), boys (N = 62) and girls. 95% bias corrected and accelerated bootstrap confidence intervals are given in square brackets in the upper half of the table. * denotes p < .05, ** < .01, and *** < .001, † indicates p = .05. Significant correlations are given in bold. No corrections were made for multiple correlations.

	1	2	3	4	5	6	7	8	9	10	11	12
**Total sample**												
1 COBEL total		**[.544; .768]**	**[.627; .810]**	**[.804; .902]**	[-.146; .209]	[-.100; .251]	[-.008; .320]	[-.097; .249]	[-.022; .321]	[-.095; .269]	[-.301; .044]	[-.259; .101]
2 COBEL food component	**.670*****		**[.144; .467]**	**[.270; .570]**	[-.176; .215]	[-.173; .210]	[-.195; .157]	[-.170; .164]	[-.124; .242]	[-.187; .162]	[-.288; .060]	[-.232; .141]
3 COBEL social component	**.731*****	**.312*****		**[.232; .558]**	[-.208; .169]	[-.190; .157]	**[.028; .361]**	[-.156; .176]	**[.006; .389]**	**[.049; .368]**	[-.227; .099]	[-.186; .183]
4 COBEL environmental component	**.859*****	**.428*****	**.411*****		[-.128; .199]	**[-.004; .329]**	[-.083; .258]	[-.071; .310]	[-.121; .235]	[-.185; .212]	[-.311; .036]	[-.284; .064]
5 Surgency	.023	.021	-.025	.029		[-.295; .057]	**[-.479; -.112]**	[-.207; .145]	[-.045; .283]	[-.127; .250]	[-.292; .044]	[-.232; .108]
6 Negative Affectivity	.078	.022	-.015	**.176***	-.117		**[-.412; -.053]**	**[.045; .387]**	[-.241; .112]	[-.116; .266]	[-.178; .140]	[-.296; .025]
7 Effortful Control	.156	-.026	**.198***	.090	**-.311*****	**-.248****		[-.211; .135]	[-.042; .298]	[-.181; .169]	[-.110; .226]	[-.047; .312]
8 Age	.083	-.002	.012	.123	-.027	**.218***	-.045		**[.057; .395]**	[-.269; .952]	**[.076; .970]**	[-.250; .114]

	1	2	3	4	5	6	7	8	9	10	11	12
9 Verbal Fluency	.146	.069	**.200***	.057	.119	-.065	.134	**.232****		[-.254; .092]	[-.068; .269]	**[.017; .343]**
10 Parenting Style - Authoritarian	.090	-.016	**.214***	.014	.065	.077	-.002	-.093	-.094		[-.272; .058]	[-.118; .244]
11 Parenting Style - Permissive	-.128	-.116	-.062	-.132	-.117	-.016	.062	**.237****	.104	-.108		[-.342; .028]
12 Parenting Style - Authoritative	-.077	-.055	.003	-.109	-.055	-.140	.133	-.059	**.185***	.055	-.159	

	1	2	3	4	5	6	7	8	9	10	11	12
**Boys**												
1 COBEL total		**[.557; .844]**	**[.500; .828]**	**[.811; .917]**	[-.316; .188]	**[-.042; .504]**	[-.269; .241]	[-.325; .220]	[-.386; .188]	[-.171; .357]	[-.339; .162]	[-.493; .024]
2 COBEL food component	**.724*****		**[.122; .574]**	**[.250; .672]**	[-.256; .275]	[-.099; .437]	[-.367; .164]	[-.318; .213]	[-.280; .241]	[-.257; .281]	[-.371; .177]	[-.405; .127]
3 COBEL social component	**.689*****	**.359****		**[.121; .619]**	[-.354; .183]	[-.212; .393]	[-.245; .295]	[-.308; .203]	[-.206; .351]	[-.009; .450]	[-.255; .181]	[-.359; .189]
4 COBEL environmental component	**.875*****	**.486*****	**.386****		[-.333; .177]	**[.013; .487]**	[-.269; .240]	[-.291; .235]	[-.520; .063]	[-.220; .340]	[-.355; .159]	**[-.540; -.042]**
5 Surgency	-.081	.008	-.090	-.089		[-.412; .122]	**[-.558; -.077]**	[-.074; .385]	[-.092; .405]	[-.263; .282]	[-.277; .229]	[-.183; .305]
6 Negative Affectivity	.247†	.185	.092	**.264***	-.144		**[-.558; -.107]**	[-.083; .376]	**[-.527; .022]**	[-.141; .362]	[-.436; .039]	[-.390; .102]
7 Effortful Control	-.019	-.122	.033	-.015	**-.341****	**-.348****		[-.439; .039]	[-.130; .414]	[-.197; .250]	[-.048; .453]	[-.202; .290]
8 Age	-.037	-.052	-.046	-.010	.163	.152	-.208		[-.038; .464]	[-.339; .184]	**[.045; .521]**	[-.381; .152]

	1	2	3	4	5	6	7	8	9	10	11	12
9 Verbal Fluency	-.102	-.008	.080	-.235	.164	**-.269***	.143	.223		[-.369; .196]	[-.218; .236]	**[.148; .558]**
10 Parenting Style - Authoritarian	.095	.014	.234	.057	-.006	.104	.034	-.080	-.107		[-.347; .124]	[-.249; .307]
11 Parenting Style - Permissive	-.094	-.084	-.041	-.101	-.034	-.207	.215	**.301***	.008	-.111		[-.446; .081]
12 Parenting Style - Authoritative	-.241	-.141	-.090	**-.293***	.075	-.159	.060	-.113	**.366***	.010	-.177	

	1	2	3	4	5	6	7	8	9	10	11	12
**Girls**												
1 COBEL total		**[.398; .757]**	**[.583; .815]**	**[.714; .910]**	[-.080; .403]	[-.290; .237]	[-.134; .339]	[-.010; .468]	**[.109; .504]**	[-.134; .292]	[-.393; .098]	[-.219; .262]
2 COBEL food component	**.609*** **		[-.026; .428]	**[.073; .575]**	[-.189; .323]	[-.395; .120]	[-.293; .198]	[-.184; .309]	[-.110; .373]	[-.260; .165]	[-.401; .066]	[-.217; .265]
3 COBEL social component	**.719*** **	.219		**[.123; .558]**	[-.155; .338]	[-.334; .193]	[-.033; .431]	[-.171; .327]	**[-.006; .512]**	[-.009; .448]	[-.345; .195]	[-.235; .254]
4 COBEL environmental component	**.831*** **	**.344** **	**.367** **		[-.060; .390]	[-.118; .371]	[-.240; .246]	**[.038; .488]**	**[.060; .461]**	[-.255; .203]	[-.371; .108]	[-.222; .281]
5 Surgency	.176	.059	.086	.184		[-.373; .090]	**[-.534; -.022]**	[-.453; .050]	[-.102; .366]	[-.159; .350]	[-.376; .102]	[-.392; .088]
6 Negative Affectivity	-.018	-.134	-.074	.123	-.135		[-.424; .129]	**[.046; .504]**	[-.084; .406]	[-.224; .289]	[-.074; .362]	[-.329; .150]
7 Effortful Control	.110	-.053	.222	-.001	**-.286***	-.139		[-.103; .400]	[-.247; .235]	[-.333; .208]	[-.283; .161]	[-.165; .372]
8 Age	.226	.052	.083	**.266***	-.218	**.291***	.166		**[.020; .495]**	[-.343; .131]	[-.050; .428]	[-.226; .302]
9 Verbal Fluency	**.313** **	.132	**.251***	**.274***	.122	.170	-.004	**.253***		[-.320;	[-.080;	[-.303;

	1	2	3	4	5	6	7	8	9	10	11	12
										.200]	.408]	.260]
10 Parenting Style - Authoritarian	.087	-.039	.226	-.032	.102	.033	-.049	-.114	-.068		[-.323; .171]	[-.190; .347]
11 Parenting Style - Permissive	-.158	-.158	-.080	-.146	-.158	.154	-.063	.197	.173	-.078		[-.389; .097]
12 Parenting Style - Authoritative	.019	.024	.022	.027	-.169	-.101	.102	.040	-.019	.089	-.146	
